# GAD‐65 antibodies in a case of HNF1A‐Maturity‐Onset Diabetes of the Young: Double diabetes?

**DOI:** 10.1002/ccr3.4151

**Published:** 2021-05-04

**Authors:** Åke Sjöholm

**Affiliations:** ^1^ Division of Endocrinology and Diabetology Department of Internal Medicine Gävle Hospital Gävle Sweden; ^2^ University of Gävle Gävle Sweden

**Keywords:** autoimmunity, diabetes, GAD‐65, MODY

## Abstract

Diabetes classification is not as defined as it used to be. A patient with one type of diabetes can have diagnostic criteria of another type, which may affect the course of the disease. Clinicians need to consider that when dealing with patients who do not fit the exact description of their diagnosed type of diabetes.

## Graphical abstract

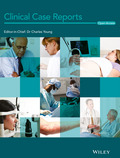

## 1 INTRODUCTION

In daily clinical practice, we are inclined to classify the type of diabetes individual patients have as one type. A patient with HNF1A‐MODY and also positive for GAD‐65 autoantibodies is presented.

In clinical practice, diabetes is mainly categorized into two main types, type 1 diabetes (T1D) or type 2 diabetes (T2D), along with some more rare or uncommon forms (see below).

Type 1 diabetes is an autoimmune disease with the presence of autoantibodies, *i.a*. against the insulin‐producing β‐cell antigen GAD‐65, which eventually leads to insulinopenia due to rapid destruction of β‐cells. Type 1 diabetes was previously called juvenile diabetes based on the incidence of the disease being highest at these ages. However, T1D may unfold at any age.

Type 2 diabetes is more of a cardiovascular lifestyle disease where lack of exercise and excess calories lead to visceral obesity with concomitant insulin resistance that in genetically predisposed individuals result in overt diabetes. Type 2 diabetes was previously called elderly onset diabetes because the prevalence increases with increasing age. However, this designation is now completely misleading. In the past T2D has also been called noninsulin‐dependent diabetes, an epithet that is also inappropriate as at least 30% of patients with T2D sooner or later need supplemental therapy with insulin in some form due to a progressive loss of β‐cells.

“Latent Autoimmune Diabetes in Adults” (LADA) can be described as a slowly progressing T1D in slightly older individuals with gradual autoimmune destruction of their β‐cells. As noted recently, LADA patients are oftentimes misclassified as T2D and for several years treated with oral agents with decreasing efficacy over time.^1^

In addition to these groups, there are monogenic types of diabetes, *for example,* various forms of "Maturity‐Onset Diabetes of the Young" (MODY).^2^, ^3^

According to the current diagnostic criteria, MODY is characterized by the absence of autoantibodies to β‐cell antigens.^2^, ^3^, ^4^ If you are antibody positive, it follows you cannot have MODY. But is it really that simple?

As the molecular genetics gets more refined, easily accessible and affordable, it has become increasingly clear that one can have not just one type of diabetes but at least two simultaneously or consecutively.

## 2 CASE PRESENTATION

The patient is a 73‐year‐old man of Finnish ethnicity with onset of diabetes, considered T2D, at the age of 30. He has a mild sensory diabetic neuropathy and moderate bilateral retinopathy. At diagnosis, he was started on metformin and approximately 20 years later insulin was added. For unknown reasons, he was never on sulfonylureas.

Strikingly many relatives on the maternal side have/had diabetes of a type‐2‐like form, which resulted in the patient being diagnosed with hepatocyte nuclear factor 1‐alpha (HNF1A)‐MODY in 2016 and being referred from primary care to our hospital for specialist care. Also the patient's daughter is heterozygous for the pathogenic mutation HNF1A:c.872dupC,p. Gly292ARgfs*25, which is the same mutation the father carries.^4^

The patient also has a well‐controlled hypertension and a chronic normocytic anemia, and has stented a left‐sided renal artery stenosis. His BMI was 27 kg/m^2^.

His medication consisted at first visit of NovoMix 30 in a dose of 25 U b.i.d. (0.62 U/kg), metformin 850 mg b.i.d., acetyl salicylic acid 75 mg q.d., doxazosin 4 mg q.d., metoprolol 50 mg b.i.d., felodipine 10 mg q.d., and losartan 100 mg q.d. The patient's HbA1c was 56 mmol/mol (7.3% DCCT), and—surprisingly—he had positive titers of IgG antibodies against the β‐cell antigen GAD‐65 (449 U/mL [ref. <5]) but not against the IA‐2 antigen. The patient's endogenous insulin production was suboptimal as the level of C‐peptide only increased from 0.22 to 0.33 nmol/L (0.66‐1.0 ng/mL) after meal stimulation.

The autoimmunity was discovered more or less by chance; the junior resident at the hospital outpatient clinic ordering the laboratory work up being oblivious of the view that MODY patients by definition do not have diabetes‐associated antibodies.

The patient was started on dulaglutide 1.5 mg q.w., an agonist for the glucagon‐like peptide 1 (GLP‐1) receptor with potent antidiabetic effects. Glucagon‐like peptide 1 has also potentially trophic and antiapoptotic effects on β‐cells (at least in animal models and in human β‐cells ex vivo^5^) and improves β‐cell glucose sensitivity ^6^ without risk of hypoglycemia or weight gain. Glucagon‐like peptide 1‐based therapy has been shown to work well in HNF1A‐MODY.^7^, ^8^, ^9^ The classic treatment of HNF1A‐MODY, however, has for many years been sulfonylurea drugs, also with good results but with a risk of hypoglycemia and weight gain. The patient's insulin regimen was maintained because of his low C‐peptide levels, while metformin was discontinued. Four months later, the patient's HbA1c, body weight, and insulin dose were largely unchanged. The combination tablet Synjardy (empagliflozin 5 mg + metformin 850 mg) b.i.d. was added to improve glycemia and 3 months later the HbA1c was 49 mmol/mol (6.6% DCCT).

The patient's daughter with HNF1A‐MODY was, unlike her father, negative for autoantibodies to GAD‐65, IA‐2, ZnT8, ICA and insulin. The patient himself was not tested for autoantibodies to ZnT8, ICA and insulin.

Patients with HNF1A‐MODY have an increased risk of adenomatosis of the liver.^10^ However, ultrasound examination of the liver showed no abnormality.

## 3 DISCUSSION

### 3.1 HNF1A‐MODY

Maturity‐Onset Diabetes of the Young accounts for only 3%‐4% of all diabetes in the Western world. HNF1A‐MODY (previously called MODY‐3) is a predominant form of monogenic diabetes and is caused by mutations in the transcription factor HNF‐1α.^11^, ^12^ Because HNF‐1α is very important for the β‐cell functional differentiation and growth as well as its ability to synthesize and release insulin, mutations in this transcription factor lead to defective insulin secretion.^12^ However, it often takes a long time before overt diabetes is diagnosed, as mutations in HNF‐1α compensatory downregulate the expression of SGLT‐2 in the kidneys.^4^ This causes a lowered kidney threshold with increased glucosuria, which lowers glycemia. The same mechanism is used by the latest class of antidiabetic drugs, SGLT‐2 inhibitors.

### 3.2 Double diabetes

The patient in this paper shows evidence of two types of diabetes, a congenital monogenic type (HNF1A‐MODY) and an acquired autoimmune type directed against a known β‐cell antigen (GAD‐65). This is an unexpected and surprising finding, as HNF1A‐MODY and autoimmune diabetes do not have much pathogenic mechanisms in common in β‐cell dysfunction. The mutation in HNF‐1α results in defective insulin secretion whereas the autoimmune process results in apoptotic cell death, two completely different mechanisms. The extent to which the autoimmune process has contributed to the patient's poor insulin production is impossible to say but will be monitored over time and we are also evaluating whether GLP‐1‐based therapy can slow down or reverse this. It is likely, however, that he will need to continue with insulin in some form. A few (1%‐2%) of nondiabetic people has GAD‐65 antibodies without ever getting diabetes. However, antibody titers in such cases are usually much lower than the level in the current case.

It is very difficult to prove if, or to what extent, the autoimmunity in this patient—albeit fairly strong—has contributed to his diabetes and impaired insulin secretion. Nonetheless, in a study of 77 individuals with long‐duration HNF1A‐MODY the lowest C‐peptide level was 0.36 nmol/L,^13^ thus higher than in the present patient. This lends support to the notion that the autoimmunity contributed to the patient's impaired insulin secretion.

The coexistence of monogenic and autoimmune diabetes is unusual, but not unique. Anecdotal case reports have been published,^14^ as well as more systematic reviews.^15^, ^16^, ^17^ In a Czech material, about 30% of HNF1A‐MODY patients were also positive for antibodies to GAD‐65,^16^ whereas only 1% in a British study ^17^ and 15%‐20% in a Scandinavian report.^15^ Coexistence of biomarkers for autoimmune diabetes and types of monogenic diabetes other than HNF1A‐MODY has also been described.^15^, ^16^, ^17^ Interestingly, an SNP (rs2650000) in the gene for HNF‐1α has been shown to predispose to autoimmune diabetes.^18^

In daily clinical practice, we are inclined to classify the type of diabetes individual patients have as one type, but in recent years the concept of “double diabetes” has received increased attention in the scientific literature.^19^, ^20^, ^21^, ^22^, ^23^ This is not least due to the fact that overweight and obesity are increasingly abundant in large parts of the world and also affect patients with T1D. Of course, having T1D does not provide protection against getting abdominal obesity and consequent insulin resistance, a cardinal finding in T2D. Thus, many patients with T1D in fact also have T2D with the cardiovascular risk factor burden the latter entails. In most studies, "double diabetes" is defined as T1D combined with the metabolic syndrome. "Double diabetes" is, however, not an established concept but herein refers to diabetes which is caused by two completely different mechanisms of action. The frequency of the comorbidity varies; in a German material it was about 25% and individuals with "double diabetes" showed a greatly increased risk of both microvascular and macrovascular complications compared to T1D, independent of glucose control,^19^ finding essentially similar to that in a subgroup analysis of the DCCT study.^21^ Recently, a Swedish study showed that increasing BMI in male T1D patients is associated with an increased risk of mortality, cardiovascular disease, and heart failure.^23^ It has also been reported that obesity and insulin resistance can interact with the immune system and thus aggravate the autoimmune attack.^24^

Finally, a case description shows that one can also have triple diabetes: HNF1A‐MODY with T1D and the metabolic syndrome.^25^

The take home message of this case report is that one thing does not exclude another (or a third), something to keep in mind not least in the event of an unexpected deterioration in glycemic control.

## CONFLICT OF INTEREST

Å.S. has received lecture and consultancy fees from Boehringer‐Ingelheim, Novo‐Nordisk, MSD, Sanofi, Pfizer and Astra‐Zeneca.

## AUTHOR CONTRIBUTIONS

ÅS provided care for the patient, researched data, wrote the manuscript, and edited/reviewed the manuscript. ÅS is the guarantor of this work and, as such, had full access to all the data in the case presentation and literature review and takes responsibility for the integrity of the information presented.

## ETHICS APPROVAL

The patient gave informed consent to this publication.

## Data Availability

Data are available in the patient's medical record.
